# Revision of a Stemless Anatomic Implant into a Stemless Reverse Implant

**DOI:** 10.1155/2021/6667871

**Published:** 2021-01-11

**Authors:** Christian Schoch, Michael Dittrich, Leander Ambros, Michael Geyer

**Affiliations:** St. Vinzenz Klinik Pfronten, Department for Shoulder and Elbow Surgery, Kirchweg 15, 87459 Pfronten, Germany

## Abstract

**Introduction:**

Stemless anatomic implants are the growing standard for solving osteoarthritis of the shoulder. If there are secondary rotator cuff insufficiency and the need to revise the implant into a reverse total shoulder, there is usually the option to revise it into a stemmed implant with losing the benefits of stemless implants. There are only a few stemless reverse implants available on the market. Usually, they are recommended as primary implants, but not for revision surgery. *Case Report*. A 61-year-old male with an indwelling anatomic stemless TESS (Total Evolutive Shoulder System, Zimmer Biomet, Warsaw, USA) implant presented in our clinic with growing pain and loss of range of motion. The TESS was implanted in 2007 as a hemishoulder arthroplasty. The X-ray was showing a stable implanted corolla with clearly growing protrusion of the glenoid. Because of the clinical presentation and the ultrasound investigation that showed only remnants of the supraspinatus and infraspinatus left, we decided that it is necessary to revise the stable implant in a reverse total shoulder arthroplasty. As the TESS was not designed to be convertible, it was clear that it has to be explanted. Due to the young age of the patient, we proposed an “off-label” change to a stemless reverse implant, e.g., the LIMA SMR stemless reverse prosthesis. A revision was possible without much bone loss, so the stemless reverse implant could be used. The clinical and radiological 2-year follow-up showed a good result.

**Conclusion:**

Stemless implants are well known for anatomic implants, rarely for reverse implants, and seldom used for revision into reverse stemless. This case report shows that it is possible to revise a stemless anatomic implant with a stemless reverse, as long as the rules for implantation are applicable.

## 1. Introduction

Since the introduction of stemless anatomic shoulder arthroplasty, a lot of follow-up studies have been published and the anatomic stemless shoulder is becoming the standard procedure in patient care [1, 2]. One of the main reasons for revision of anatomic implants is secondary cuff failure, so a reverse shoulder arthroplasty is performed in most of the cases. Thinking about the reasons for stemless arthroplasty—saving bone stock—the question is if it is possible to implant a stemless reverse implant in a revision case.

Right now, there are only two stemless reverse implants in the literature with follow-up data, which are both not available on the market anymore. LIMA provides one of the rare stemless systems which are convertible or used as a primary reverse stemless implant. To our knowledge, there is no description of cases using the stemless RSA as a revision implant. Furthermore, no follow-up study for the primary use of the SMR stemless reverse is available in the PubMed database.

This report shows the revision of a TESS anatomic prosthesis with secondary rotator cuff insufficiency using a LIMA SMR stemless reverse implant, the 2-year clinical and radiological follow-up.

Shoulder arthroplasty is the accepted treatment for primary and secondary glenohumeral arthritis resulting in pain reduction and improvement of shoulder function. Due to possible intraoperative complications with standard stemmed implants, stemless designed prostheses evolved starting in 2004 with the TESS (Zimmer Biomet, Warsaw, IN, USA), and in 2007, the Eclipse (Arthrex, Naples, USA) followed. Up to now, most of the implant providers offer a stemless anatomical shoulder replacement in their portfolio. These systems aim for an option to replace the humeral head independently from the humeral axis. The clinical effect and midterm survivorship of stemless anatomic implants are well published as equal to the classic stemmed implants [2–9].

### 1.1. The Used Implants

The Zimmer Biomet Total Evolutive Shoulder System (TESS)—the first on the market in 2004—is a three-component system based upon a six-armed porous-coated metaphyseal component. This corolla-called component is impacted into the metaphyseal bone; then, the humeral head is inserted via a corresponding female morse taper.

Released in 2015, the LIMA Shoulder Modular Replacement (SMR) stemless shoulder system is a convertible system which has four parts for anatomic configuration (humeral core component, double male Morse taper, locking screw, and humeral head) and two parts for reverse configuration (humeral core component and reverse liner). The humeral core component is composed of trabecular titanium designed for bony ingrowth and is seated by impaction. When utilized in the reverse configuration, a metallic reverse liner is impacted into the humeral core component. This metallic liner then articulates with the all-polyethylene glenosphere.

The core is sized to get stability by nearly having contact to the cortical ring for additional stability. Therefore, theoretical as long as the revision of the anatomic implant leaves an intact cortical ring of the humeral bone, the implanting of the SMR stemless core is technically possible, as it would be in primary implantation.

Our hypothesis was that it is possible to revise the TESS implant with the LIMA SMR stemless reverse implant, to save the lasting bone stock in this young patient with comparable results to stem implants.

## 2. Case Report

### 2.1. Case 1

A 61-year-old male with an indwelling anatomic stemless prosthesis presented in our clinic with growing pain and loss of the ability to lift the arm above 70° of abduction and flexion.

The anatomic TESS was implanted in 2007 as a hemishoulder arthroplasty.

The X-ray was showing a stable implanted corolla with a growing loss of cartilage at the glenoid side. Protrusion was obvious up to the base of the coracoid process. Because of the clinical presentation, an ultrasound investigation was done, showing only remnants of the SSP and ISP so we decided that it is necessary to revise the stable implant in a reverse total shoulder arthroplasty. As the TESS was not designed to be convertible, it was clear that it has to be explanted. Due to the young age of the patient, we proposed a change to a still stemless construct, e.g., the LIMA SMR stemless reverse, if possible.

### 2.2. OR Procedure

**Figure 1:**
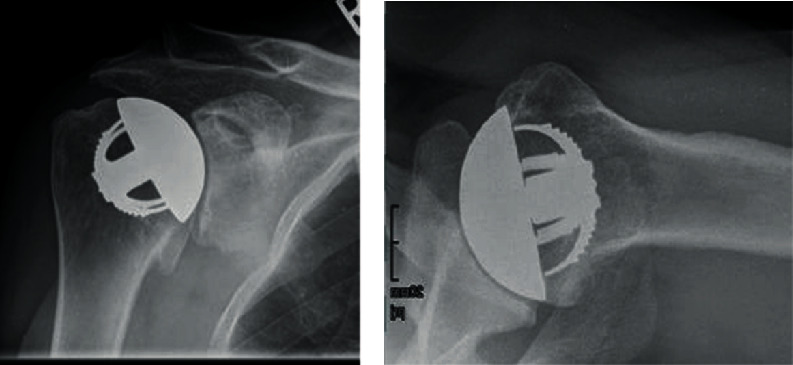
Preoperative X-ray, indwelling TESS anatomical stemless implant.

Figure 1 shows the pre-op X-ray.

**Figure 2:**
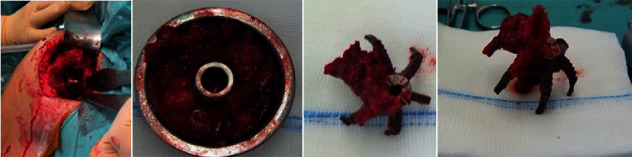
Corolla in situ, back of the humeral head, and corolla explanted.

**Figure 3:**
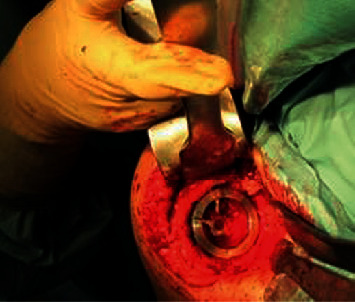
Seated core of the SMR.

**Figure 4:**
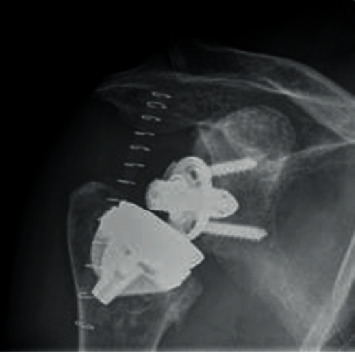
First post-op X-ray.

Under interscalene block and anesthesia, implant removal was done using the deltopectoral approach. The glenoid was worn, supraspinatus was completely gone, the anterior 2/3 of the infraspinatus were gone, and the subscapularis was partially torn, with teres minor still intact. After disconnecting the humeral head component from the corolla, it was easily possible to loosen the implant by using a small chisel along the arms of the corolla (Figure 2). Performing this very gently, there was enough bone stock left for the SMR stemless implant. We used the adherent spongeous bone from the prosthesis arms for “impaction grafting” and used a maximum-sized core implant to get good primary fixation of the core (Figure 3). Therefore, we decided to go for stemless and do the implantation of the glenoid component first and then perform the implantation of the original implant on the humeral side. In the OR, the functional ROM was good and there was no sign of instability or impingement. Wound closure was done in a standard fashion; an abduction pillow was used for 4 weeks after the operation, but the patient was allowed to use his arm/hand as long as it was pain-free possible. A post-op X-ray ap was done (Figure 4).

**Figure 5:**
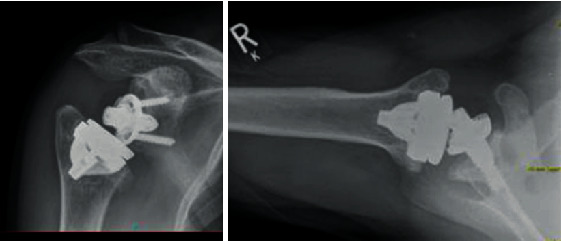
Eight weeks post-op.

**Figure 6:**
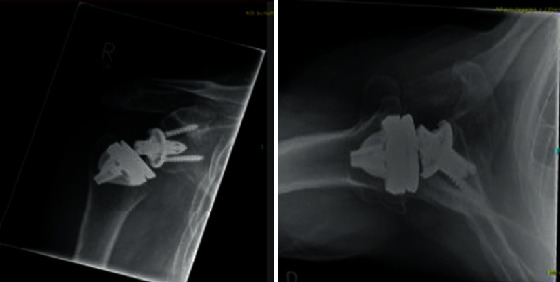
Two-year control.

After 8 weeks, the X-rays showed bony integration (Figure 5). There were no signs of loosening; even in the 2-year control, there was no radiolucent line (Figure 6).

The constant score [10] did improve significantly from pre-op 28 to 78 after 2 years. The detailed score is shown in Table 1.

The patient was very satisfied with the functional results and reduction of pain. He was even able to start renovating his house 9 weeks after the operation.

## 3. Discussion

The goal of our case report was to show that even a revision from one stemless implant into another stemless reverse implant is possible and leads to good functional and radiological results and reduction of pain.

One of the most common failures of anatomic shoulder arthroplasty is secondary rotator cuff insufficiency. Melis et al. [11, 12] showed in a failure analysis of 37 patients that 24 patients had undergone revision into reverse shoulder arthroplasty after a mean interval of 75.3 months after implantation of an anatomic arthroplasty. Habermeyer et al. [13] showed a cranial migration in 14.7% after 9 years; Young et al. showed 46.5% cranial head migration after a mean follow-up of 10 years [14]. Although Habermeyer et al. showed that a secondary cranialization of the humeral head does not necessarily lead to a revision of RSA, the cuff deficiency leads to worsening of the clinical result.

Because of the impression of having a bone-saving option, stemless anatomic shoulder arthroplasties are mostly performed in younger patients. Actual studies show survival rates up to 93% in 10 years for stemless TSA [15]. But the younger the patients are, these rates get as low as 83% survivorship after 10 years for a stemless anatomic implant [16].

The younger patients have the higher the risk of revision surgery in their lifetime—or even for multiple revisions.

The idea of our procedure was to save as much bone stock as possible for the revision of the revision. Thus, looking at the options on the market, there is stemless, short-stem, or standard stem reverse arthroplasty.

If the anatomic stemless implant was used because of problems with the humeral shaft (e.g., posttraumatic deformation), it would be a big advantage if stemless revision would be possible.

All actually published studies on stemless reverse shoulder arthroplasty are showing the results of the Biomet TESS implant. According to Upfill-Brown et al. [17], all those studies show good results in the clinical scores and radiological outcome. For the LIMA SMR stemless implant, no clinical results are published up to now.

Naturally, our case report has some inherent limitations.

First of all, it is a case report showing the technical feasibility of the operation. There is no data of the used implant for comparison of the results in the literature.

With very good clinical results, the clinical outcome of our patient is comparable to the outcome of primary reverse arthroplasty or revision surgery [2–9, 14–17]. The radiological results show a good short-term follow-up; the long-term survivorship has to be waited for. With good bony integration, comparable longevity as shown in anatomic stemless studies is suggested.

## 4. Conclusion

Revision of a stemless anatomic implant into a stemless reverse implant is technically possible. The patient benefits in pain relief and function. In the midterm, there is no radiological loosening.

## Figures and Tables

**Table 1:** Constant score of the patient (including its subcategories).

	Pain	ADLs	Mobility	Strength	Overall
Pre	4	6	10	8	28
8 weeks	10	12	20	12	54
1 year	14	16	28	18	76
2 years	15	16	30	17	78
